# Transmission of Scarlatina

**Published:** 1890-02

**Authors:** 


Transmission of Scarlatina Through Circulating Libraries.—*Semaine Med.* relates that an English physician has informed the authority of a well authenticated case of transmission of scarlatina through a book taken from a circulating library. He points out that the circulation of such books is an easy means of spreading contagious matters, because infectious particles are very liable to stick to books, and because convalescents are very fond of reading. For this reason, the English authority has decided that persons affected with contagious diseases who take books from a circulating library, thereby facilitating the dissemination of the disease, are guilty of a misdemeanor, and amenable to law.—*Pharmaceutische Post.*

